# Identification of the Sfp-Type PPTase EppA from the Lichenized Fungus *Evernia prunastri*

**DOI:** 10.1371/journal.pone.0145624

**Published:** 2016-01-19

**Authors:** Olivia Schimming, Imke Schmitt, Helge B. Bode

**Affiliations:** 1 Fachbereich Biowissenschaften, Goethe Universität Frankfurt, Max-von-Laue-Str. 9, 60438, Frankfurt am Main, Germany; 2 Senckenberg Biodiversity and Climate Research Centre BiK-F, Senckenberganlage 25, 60325, Frankfurt am Main, Germany; University of Wisconsin, UNITED STATES

## Abstract

In the last decades, natural products from lichens have gained more interest for pharmaceutical application due to the broad range of their biological activity. However, isolation of the compounds of interest directly from the lichen is neither feasible nor sustainable due to slow growth of many lichens. In order to develop a pipeline for heterologous expression of lichen biosynthesis gene clusters and thus the sustainable production of their bioactive compounds we have identified and characterized the phosphopantheteinyl transferase (PPTase) EppA from the lichen *Evernia prunastri*. The Sfp-type PPTase EppA was functionally characterized through heterologous expression in *E*. *coli* using the production of the blue pigment indigoidine as readout and by complementation of a *lys5* deletion in *S*. *cerevisiae*.

## Introduction

Lichen-forming fungi are symbiotic organisms forming associations with green algae, cyanobacteria, or both [1]. They synthesize a plethora of natural products, many of which possess biological activities [2]. Extracts from whole lichens showed antimicrobial, anti-inflammatory, analgesic or cytotoxic activity [3–6], rendering lichens interesting organisms for pharmaceutical applications [7,8]. One of the ecological functions of lichen compounds is damage prevention from sunlight [9]. Anthraquinones or xanthones act as strong ultraviolet filters, which could prove beneficial for the development of novel sunscreens protecting from harmful UVA rays [9]. The majority of natural products are synthesized by the mycobiont [10]. Still, the photobiont contributes to the natural product profile, often lichenizing fungi do not produce biological active molecules without their suitable algal partner [10].

The slow growth and the difficulty to establish pure cultures of the mycobiont hamper experimental approaches to study lichens [11]. Moreover, there exist few direct transformation systems [12],and many genes remain silent under laboratory conditions [13,14]. For this reason, heterologous expression of lichen genes in surrogate hosts is a sought-after approach [11,15]. Promising hosts could be *Escherichia coli* and *Saccharomyces cerevisiae* with established transformation systems and vectors [14,16].

While up to 1000 substances from lichens are known to date [17], only a few have been characterized in detail for their biological and therapeutic potential. Prominent representatives are the anthraquinone parietin [18] having antifungal activity [19] and the dibenzofuran usnic acid [20] showing anti-inflammatory activity in tests with rats [21]. Most of the typical lichen compounds are polyketides synthesized by polyketide synthases (PKS). However, other natural products may have gone unnoticed due to low concentrations in the lichen thallus. Especially natural products derived from non-ribosomal peptide synthetases (NRPS) have remained unexplored.

So far, all fungal and bacterial NRPS as well as type I and type II PKS characterized to date require a phosphopantetheinyl transferase (PPTase) to post-translationally attach the 4’-phosphopantetheine (Ppant) arm from CoA to the serine residue of the thiolation domain [22,23]. Three main types of PPTases are known: Sfp-type, AcpS-type, and integrated PPTases. The Sfp-type PPTase is generally regarded to be associated with secondary metabolism. Sfp-type PPTases are approximately 240 aa long and have a monomeric structure [24]. The name is derived from Sfp which is associated with the surfactin biosynthesis gene cluster and was discovered first in *Bacillus subtilis* [25]. By contrast, the AcpS-type is only half the size of the Sfp-type and mainly associated with fatty acid biosynthesis (FAS) [26]. However, integrated PPTases are located within the fatty acid synthases like the type I fatty acid synthase from yeast [27] and in several PKS from plants and bacteria [28].

Besides playing a part in the secondary metabolism Sfp-type PPTases can also act in the primary metabolism such as in the lysine biosynthesis or FAS if there is no AcpS-type PPTase. [28]. Moreover, due to its low substrate specificity it accepts various CoA analogs including covalently attached reporter molecules like affinity or fluorescent tags and thus can be used as a biochemical toolbox or to label specific protein tags [29,30].

Sfp-type PPTases are found across all three domains of life so that the type was additionally grouped into the subclasses F/KES and W/KEA to specify its function. Apart from PPTases involved in polyketide, glycolipid and lysine biosynthesis as well as eukaryotic PPTases, Sfp belongs to the subtype W/KEA [28].

In this work, we identified a Sfp-type PPTase EppA from the lichenizing fungus *Evernia prunastri*. Furthermore, we expressed *eppA* heterologously in *E*. *coli* and *S*. *cerevisiae* to test its functionality by indigoidine production and complementation of *lys5* deletion, respectively.

## Material and Methods

### Strains and culture conditions

All strains are listed in S1 Table. All *E*. *coli* strains were cultivated in LB media [31]. For solid media 1.5% (w/v) agar was added. *S*. *cerevisiae* CEN.PK2-1C and derivatives were cultured in YEPD media [32]. Uracil auxotroph yeast expression strains were grown in SC media [33] with 2% (v/v) glucose (SD-ura) or galactose (SG-ura), respectively. Solid media additionally contained 2% (w/v) agar. Ampicillin (100 μg/ml), chloramphenicol (20 μg/ml), kanamycin (50 μg/ml) and G418 (200 μg/ml) were used as selection markers. All strains were cultivated at 30°C.

### General molecular methods

Polymerase chain reaction (PCR) was performed using oligonucleotides obtained from Eurofins Genomics (S3 Table). Fragments with homology arms were amplified in a two-step PCR program using Phire Hot Start II DNA polymerase (Thermo Scientific) according to the manufacturers’ instructions. DNA purification was performed using MinElute PCR Purification Kit (Qiagen). Transformation of *S*. *cerevisiae* cells was done by standard LiAc transformation.

### Construction of pCK_*eppA*

For plasmids used in this study see S2 Table. The plasmid pCK_*eppA* was constructed on the basis of pCK_*mtaA*. The identified Sfp-type PPTase gene *eppA* was amplified from the genomic DNA of *E*. *prunastri* using the oligonucleotides OS_ck_eppA_for/OS_ck_eppA_rev. The vector pCK was amplified from the template pCK_*mtaA* with the oligonucleotides OS_ck_for/OS_ck_rev. The 1007 bp PCR product of *eppA* contained 40 bp homology sequences to either side of the amplified vector pCK. Both fragments were cloned by Gibson assembly [34,35].

### Construction of pYES260_*npgA* and pYES260_*eppA*

For the complementation of the *lys5* deletion *npgA* and *eppA* were cloned into the linearized vector pYES260 by yeast homologous recombination [36]. The gene *npgA* which originates from *Aspergillus nidulans* was amplified with the oligonucleotides OS_pYnpgA_for/OS_pYnpgA_rev taking pET28a_*npgA* as template. The gene *eppA* was directly taken from the genomic DNA of *E*. *prunastri* using the oligonucleotides OS_pYeppA_for/OS_pYeppA_rev.

### Deletion strain *S*. *cerevisiae* CEN.PK2-1C*∆lys5*

The construction of the *S*. *cerevisiae* strain CEN.PK2-1C*∆lys5* was carried out by substituting *lys5* for a loxP-kanMX-loxP cassette. The deletion cassette was amplified from the plasmid pUG6 [37] with the oligonucleotides OS_SClys5_for/OS_SClys5_rev. The 1666 bp large PCR product contained 40 bp homologous sites upstream as well as downstream of *lys5* and was cloned into *S*. *cerevisiae* CEN.PK2-1C via yeast homologous recombination. The chromosomal deletion of *lys5* had been verified by PCR with the oligonucleotides OS_SClys5_a1/ OS_SClys5_a4 binding downstream and upstream of the gene, respectively. Furthermore, oligonucleotides OS_SClys5_a2 and OS_SClys5_a3 having their binding site within *lys5* as well as OS_SClys5_k2 and OS_SClys5_k3 having their binding site in the introduced marker cassette have been used in combinations with the former ones to confirm the chromosomal deletion of *lys5*.

### Indigoidine production in *E*. *coli* DH10B and spectroscopic analysis

The functional characterization of *mtaA* and *eppA* was carried out by heterologous expression in *E*. *coli* using the blue pigment indigoidine as reporter. The NRPS gene *indC* responsible for the production of indigoidine is located on the plasmid pUC18_*indC* and co-expressed with the PPTase genes from the plasmids pCK_*mtaA* and pCK_*eppA*, respectively [38,39]. The experiment was carried out on solid and liquid media.

Indigoidine was extracted from 10 ml expression cultures containing Amberlite^®^ XAD-16. The harvested resin was washed with water to reduce impurities and the following extractions were carried out with one volume of methanol for 30 min at room temperature. After filtration and evaporating to dryness under reduced pressure the extracts were dissolved in 1 ml DMSO and the absorbance of the biological triplicates was measured at 595 nm [40,41].

### Complementation of the *lys5* deletion in *S*. *cerevisiae* CEN.PK2-1C

The functional characterization of *npgA* and *eppA* was carried out by complementation of the *lys5* deletion in *S*. *cerevisiae* CEN.PK2-1C as described previously [42]. The cells were transformed with the expression plasmids pYES260_*npgA* and pYES260_*eppA*, respectively. The complementation experiment was carried out on SD-ura media containing lysine and SG-ura media without lysine. Through the carbon source the gene expression of pYES260_*npgA* and pYES260_*eppA* is regulated via the inducible promoter *P*_*GAL1*_.

### Construction of PPTase phylogeny

Amino acid sequences of PPTases (listed in S4 Table) were aligned using ClustalW before a maximum likelihood phylogeny was constructed using the PhyML feature built into Geneious (v6.1.8). Branch formation was supported by bootstrapping (n = 100).

## Results

We are interested in the heterologous production of lichen natural products as sustainable contribution to the development of novel bioactive natural products. Here our main interest focuses on compounds derived from NRPS and type I PKS. So far, all NRPS and type I PKS known to date require a PPTase that modifies their thiolation domain from the inactive *apo* to the active *holo* form [43]. The PPTase catalyzes the covalent attachment of the Ppant arm obtained from CoA to the serine residue of the thiolation domain. The identification of the first PPTase from a lichen-forming fungus was managed through BLAST search of the genomic DNA from *E*. *prunastri* (unpublished data) against other PPTases. For the *in silico* search in the genome we have chosen EntD from *E*. *coli* [44], Lys5 from *S*. *cerevisiae* [45], meAcpS from *Micromonaspora echinospora* spp. [46], AcpS as well as Sfp from *B*. *subtilis* [25,47] and NpgA from *A*. *nidulans* [48]. Both, Sfp and NpgA, provided the same result and led to a gene with a length of 927 bp. All other sequences resulted in no hit.

**Fig 1:**
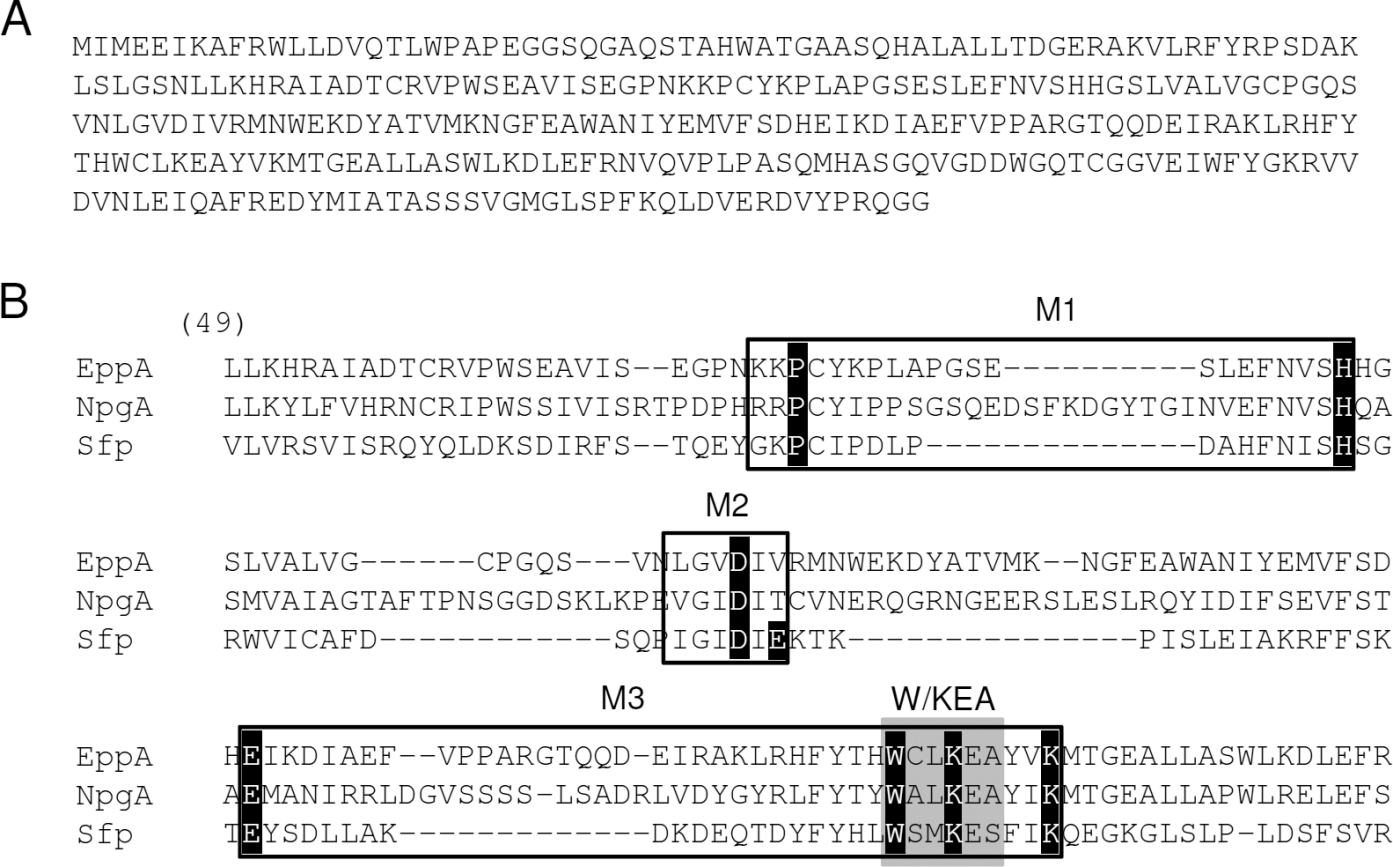
Amino acid sequence and alignment of EppA. Amino acid sequence of EppA (**A**) and partial sequence alignment of EppA from *E*. *prunastri*, NpgA from *A*. *nidulans* and Sfp from *B*. *subtilis* starting at amino acid 49 (counting from Sfp) showing motif 1–3 (M1-M3) as well as the W/KEA motif (grey box) and the corresponding conserved residues for Sfp (marked in black) (**B**).

**Fig 2:**
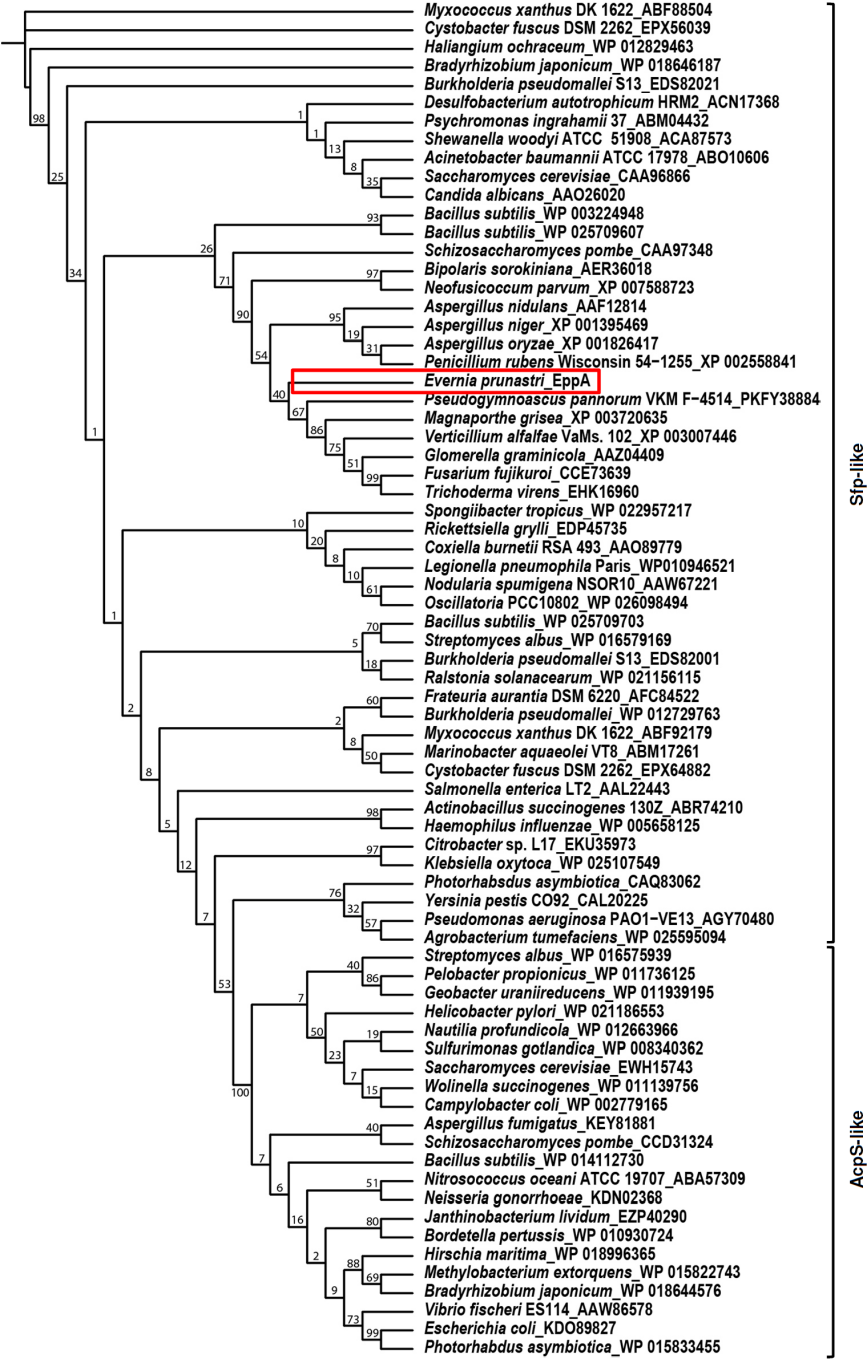
Phylogenetic tree of Sfp- and AcpS-type PPTases from different organisms. EppA (framed in red) is grouped in the Sfp-type next to PPTases from fungi and *Bacillus subtilis*. For accession numbers of sequences used see S4 Table.

**Fig 3:**
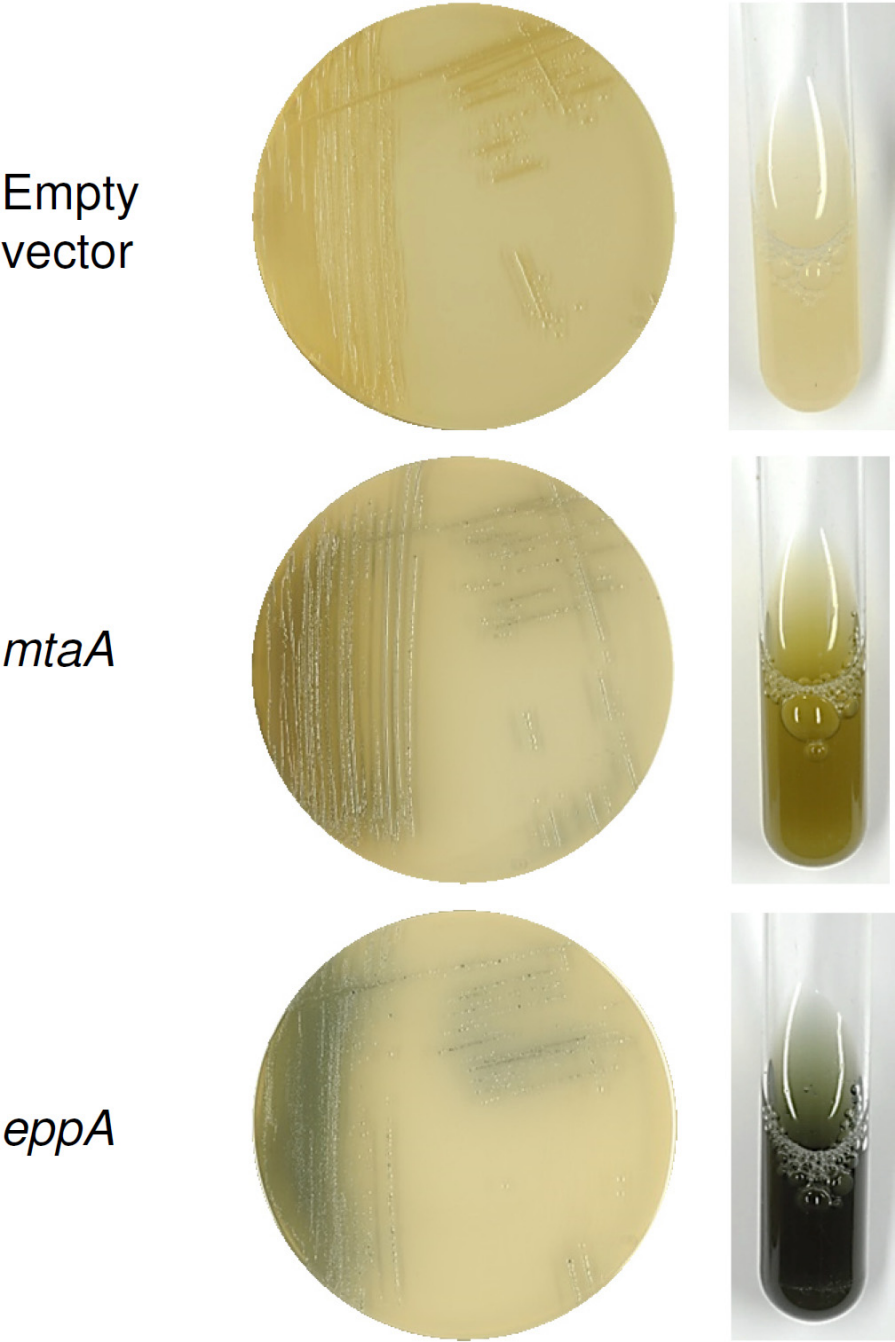
Indigoidine production assay in *E*. *coli* DH10B on solid and in liquid media. The co-expression of pUC18_indC and pACYC_tacI/I (empty vector), pCK_*mtaA* (*mtaA*) or pCK_*eppA* (*eppA*), respectively, led to blue cells for the co-expressed PPTase genes *mtaA* and *eppA*.

Due to the organism and its purpose we named the gene *eppA* (*Evernia prunastri* PPTase A; Genbank accession number: KT369532). On the basis of the BLAST search and the amino acid sequence (Fig 1), EppA could be grouped into the Sfp-type PPTases next to other Sfp-like PPTases from different fungi (Fig 2). Furthermore, the alignment of EppA, NpgA and Sfp led to the subclassification W/KEA (Fig 1). Apparently, EppA is the only PPTase encoded in the genome of *E*. *prunastri* mycobiont. Since there is no direct genetic manipulation method to test its function in the original host we chose *E*. *coli* as surrogate host. Therefore, we amplified *eppA* with homologous arms and cloned it into the linearized vector fragment pCK. The resultant plasmid pCK_*eppA* was co-expressed with pUC18_*indC*. The vector pUC18_*indC* carries the gene *indC* encoding the NRPS IndC. IndC is responsible for the cyclization and oxidation of glutamine that with air forms the blue pigment indigoidine [49]. Since the production is visual to the naked eye indigoidine is a suitable reporter for NRPS and PPTase function. The co-expression of pUC18_*indC* with pACYC_tacI/I (negative control), pCK_*mtaA* (positive control) and pCK_*eppA* resulted in the formation of blue cells for the last two constructs although cells containing *mtaA* are apparently not as much pigmented as cells expressing *eppA* (Fig 3). The PPTase encoding gene *mtaA* from the myxobacterium *Stigmatella aurantiaca* [50] was used as a positive control as it had been described to function in IndC activation [38]. The spectroscopic analysis revealed an indigoidine production of 54.8±0.6 mg/L/OD_595nm_ and 64.8±0.3 mg/L/OD_595nm_ for samples containing MtaA and EppA, respectively.

Encouraged by the functional production of EppA in *E*. *coli* its function was also tested in *S*. *cerevisiae* using a complementation test procedure according to Mootz *et al*. (2002) [42]. The PPTase Lys5 from *S*. *cerevisiae* is involved in the lysine biosynthesis as the enzymatic reduction of α-aminoadipate to α-aminoadipate semialdehyde takes place while the substrate is bound at the Ppant arm of the α-aminoadipate reductase Lys2. Thus the deletion of *lys5* results in lysine auxotrophic yeast strain that can only survive with lysine in the medium or a complementing PPTase expressed in the cell.

**Fig 4:**
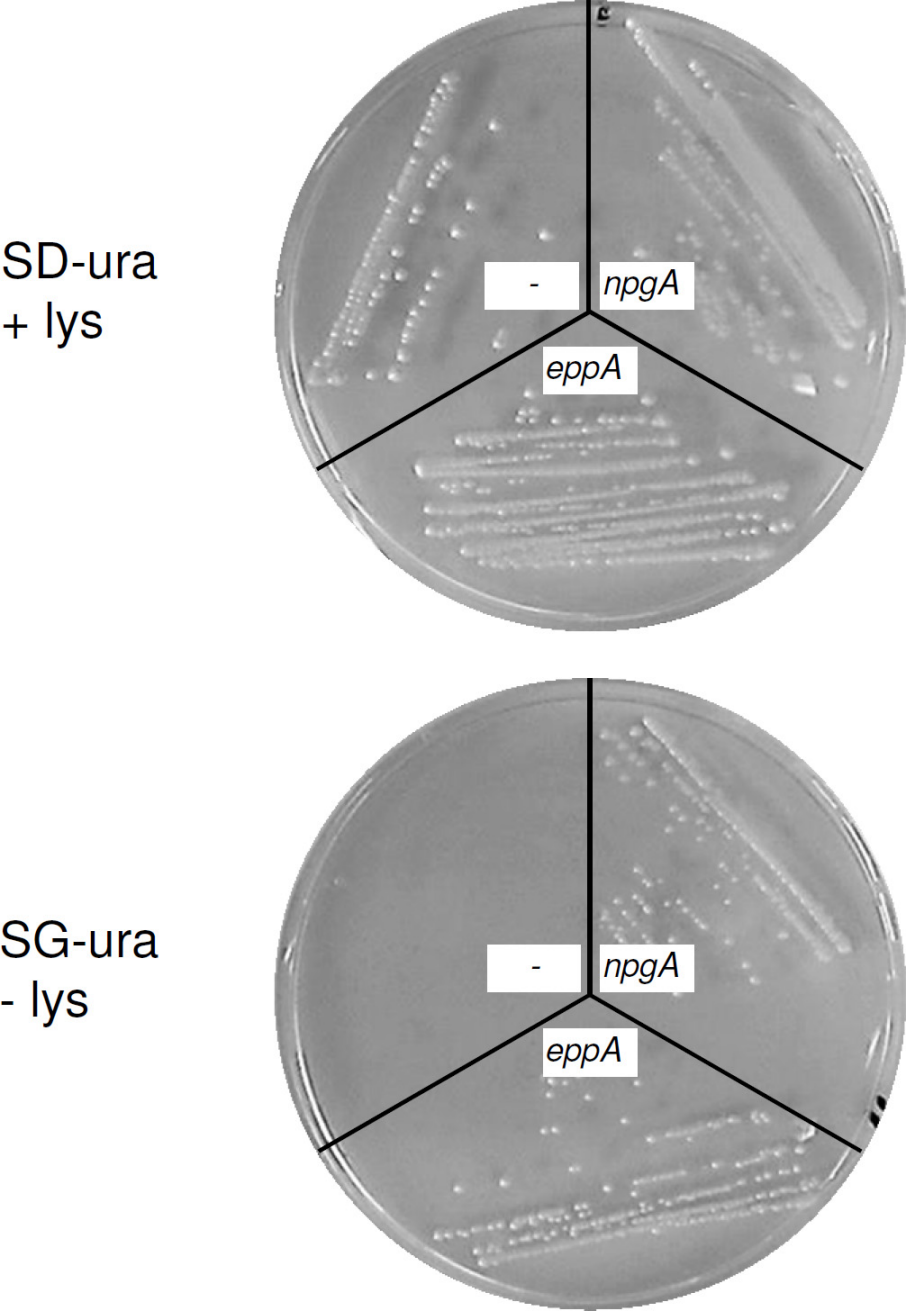
Complementation assay in *S*. *cerevisiae* CEN.PK2-1C*∆lys5* by heterologously expressed PPTase encoding genes. Constructs pYES260 (-), pYES260_npgA (*npgA*) and pYES260_eppA (*eppA*) were tested for complementation of the *lys5* deletion on SD-ura media with supplementation of lysine (SD-ura + lys) and on SG-ura media without lysine (SG-ura–lys).

The *S*. *cerevisiae* CEN.PK2-1C*∆lys5* strain was constructed by inserting a loxP-kanMX-loxP gene disruption cassette into *lys5*. Furthermore, *eppA* and *npgA* from *Aspergillus nidulans* were cloned into the expression vector pYES260, respectively. This shuttle vector carries a galactose inducible promoter *P*_*GAL1*_ which is non-induced on glucose and induced on galactose. Accordingly, yeast cells should grow independently from a complementation on SD-ura plates with added lysine. In contrast, only cells carrying a functional PPTase can grow on SG-ura plates without lysine. The complementation assay was carried out on solid media with CEN.PK2-1C∆lys5 and the corresponding expression plasmids pYES260 (negative control), pYES260_*npgA* (positive control) or pYES260_*eppA*, respectively. For the empty vector no growth could be monitored. However, cell growth could be observed for the complementation with EppA and NpgA (Fig 4).

Thus, the function of the newly identified PPTase EppA from *E*. *prunastri* could be confirmed through heterologous expression in *E*. *coli* and *S*. *cerevisiae*.

## Discussion

Natural products from lichens have gained immense interest due to their broad range of biological activity [7,8]. From *in silico* analyses of the lichen genomes [12,51–54] it is obvious that lichens harbor a yet underestimated number of PKS and NRPS encoding genes as it is also the case for fungal and bacterial natural product producers [55]. As all NRPS and type I PKS known up to date require a PPTase for activity, we initially focused on the identification of a PPTase from the lichen *E*. *prunastri* as model system that is also known as oakmoss.

The six selected PPTases used as BLAST query covered all types of known PPTases. Yet, only EppA could be identified as member of the Sfp-type PPTases related to similar PPTases from other fungi. Organisms with sole PPTases are quite common and often show a broad substrate tolerance activating several different acyl and peptidyl carrier proteins. For example PcpS from *Pseudomonas aeruginosa* can act in both primary and secondary metabolism [56].

Indigoidine production is an ideal reporter system for PPTase activity as it is highly sensitive [57]. Only a small amount of PPTase is required compared to the amount of NRPS to give the blue pigment [58]. It might be possible that the higher indigoidine production of samples containing EppA is due to a lower amount of EppA resulting from a suboptimal translation from a difference in codon usage. For the complementation of the *lys5* deletion in *S*. *cerevisiae*, yeast cell growth could be shown for the positive control NpgA and the lichen PPTase EppA. Mootz and co-workers already functionally characterized two fungal genes from *Schizosaccharomyces pombe* with this method [42]. There is no background activity–the cells either grow when complemented or not, making it a powerful tool for screening of novel PPTases from different origins [42].

Since there are no transformation methods for lichen available, the only possibility to investigate these genes is the heterologous expression in surrogate hosts like *E*. *coli*, *S*. *cerevisiae* or *Aspergillus* [14,16,59]. The expression in *Aspergillus* has the advantage that it might work independently from cDNA or the knowledge of intron/exon boundaries since the organism is able to splice introns [59]. However, *E*. *coli* and *S*. *cerevisiae* have the benefit that they are well established and a broad set of expression plasmids with different promoters and selection markers already exist [14].

So far, there are only few reports of heterologous expression of genes from lichenizing fungi [11,18]. Testing PPTases has the advantage that already poor expression is sufficient to show a product formation whereas for NRPS production and product screening much higher production of proteins is required. Up to date, NRPS production in *S*. *cerevisiae* could be shown successfully for the fungal cluster *pcbAB* from *Penicillium chrysogenum* by Siewers *et al*. (2009) involved in the production of the antibiotic precursor δ-(L-α.aminoadipyl)-L-cysteinyl-D-valine [60]. Regarding lichen genes most progress had been made by investigation of a PKS gene from *Cladonia grayi* which was heterologously expressed in *A*. *nidulans*. Two introns were successfully spliced out and RT-PCR analysis revealed the transcription of the gene under the native promoter [61].

In conclusion, a start has been made by the expression of PKS and NRPS encoding genes from lichen and the identification of the PPTase EppA but it is still a long way to produce lichen natural products in heterologous hosts.

## Supporting Information

S1 Table Strains used in this study. (DOCX)

S2 Table Plasmids used in this study. (DOCX)

S3 Table Oligonucleotides used in this study. (DOCX)

S4 Table Name and accession number of the protein sequences used in the phylogenetic tree. (DOCX)
