# Supplementary material for: Identification of the Sfp-Type PPTase EppA from the Lichenized Fungus Evernia prunastri
Source: PLoS One. 2016 Jan 19;11(1):e0145624. doi: 10.1371/journal.pone.0145624 (PMC4718654; doi:10.1371/journal.pone.0145624)
Supplement: S1 Table — (DOCX) [file pone.0145624.s001.docx]

**Supplementary table**

**S1 Table. Strains used in this study.**

| **Strain** | **Genotype** | **Reference** |
| --- | --- | --- |
| DH10B | F– *mcr*A Δ(*mrr*-*hsd*RMS-*mcr*BC) Φ80*lac*ZΔM15 Δ*lac*X74 *rec*A1 *end*A1 *ara*D139 Δ(*ara leu*) 7697 *gal*U *gal*K *rps*L *nup*G λ– | Invitrogen |
| DH10B pACYC_tacI/I | DH10B pACYC tacI/I, Cm^R^ | this work |
| DH10B pCK_*mtaA* | DH10B pCK_*mtaA*, Cm^R^ | this work |
| DH10B pCK_*eppA* | DH10B pCK_*eppA*, Cm^R^ | this work |
| CEN.PK2-1C | MATa; his3D1; leu2-3_112; ura3-52; trp1-289; MAL2-8c; SUC2 | [1] |
| CEN.PK2-1C*∆lys5* | CEN.PK2-1C*∆lys5*, G418 | this work |
| CEN.PK2-1C*∆lys5* pYES260 | CEN.PK2-1C*∆lys5* pYES260,G418, Amp^R^, Ura3 | this work |
| CEN.PK2-1C*∆lys5* pYES260_*npgA* | CEN.PK2-1C*∆lys5* pYES260_*npgA*,G418, Amp^R^, Ura3 | this work |
| CEN.PK2-1C*∆lys5* pYES260_*eppA* | CEN.PK2-1C*∆lys5* pYES260_*eppA*,G418, Amp^R^, Ura3 | this work |

**References**

1. Entian K, Kötter P. 25 Yeast Genetic Strain and Plasmid Collections. Yeast Gene Analysis - Second Edition: Elsevier; 2007. pp. 629–666.
