# Supplementary material for: Identification of the Sfp-Type PPTase EppA from the Lichenized Fungus Evernia prunastri
Source: PLoS One. 2016 Jan 19;11(1):e0145624. doi: 10.1371/journal.pone.0145624 (PMC4718654; doi:10.1371/journal.pone.0145624)
Supplement: S2 Table — (DOCX) [file pone.0145624.s002.docx]

**Supplementary table**

**S2 Table. Plasmids used in this study.**

| **Plasmid** | **Genotype** | **Reference** |
| --- | --- | --- |
| pACYC_tacI/I | p15A ori, two tacI promoters, Cm^R^ | [1] |
| pCK_*mtaA* | p15a ori, Cm^R^*, mtaA*, T7lac promoter | [2] |
| pCK_*eppA* | p15a ori, Cm^R^*, eppA*, T7lac promoter | this work |
| pYES260 | pBR322 ori, Amp^R^, 2µ ori, Ura3, GAL1 promoter, CYC1 terminator | [3] |
| pYES260_*npgA* | pYES260, *npgA* | this work |
| pYES260_*eppA* | pYES260, *eppA* | this work |
| pET28a_*npgA* | pBR322 ori, kan^R^, *npgA*, T7lac promoter | [4] |
| pUC18_indC | pUC ori, Amp^R^, *indC*, lac promoter | [5] |
| pUG6 | pBR322 ori, Amp^R^, loxP-kan^R^-loxP | [6] |

**References**

1. Lorenzen W, Ahrendt T, Bozhüyük, Kenan A J, Bode HB. A multifunctional enzyme is involved in bacterial ether lipid biosynthesis. Nat. Chem. Biol. 2014; 10: 425–427. doi: 10.1038/nchembio.1526.

2. Kegler C, Nollmann FI, Ahrendt T, Fleischhacker F, Bode E, Bode HB. Rapid determination of the amino acid configuration of xenotetrapeptide. Chembiochem. 2014; 15: 826–828. doi: 10.1002/cbic.201300602.

3. Melcher K. A modular set of prokaryotic and eukaryotic expression vectors. Anal. Biochem. 2000; 277: 109–120. doi: 10.1006/abio.1999.4383.

4. Schneider P, Bouhired S, Hoffmeister D. Characterization of the atromentin biosynthesis genes and enzymes in the homobasidiomycete Tapinella panuoides. Fungal Genet. Biol. 2008; 45: 1487–1496. doi: 10.1016/j.fgb.2008.08.009.

5. Brachmann AO, Kirchner F, Kegler C, Kinski SC, Schmitt I, Bode HB. Triggering the production of the cryptic blue pigment indigoidine from Photorhabdus luminescens. J. Biotechnol. 2012; 157: 96–99. doi: 10.1016/j.jbiotec.2011.10.002.

6. Güldener U, Heck S, Fielder T, Beinhauer J, Hegemann JH. A new efficient gene disruption cassette for repeated use in budding yeast. Nucleic Acids Res. 1996; 24: 2519–2524.
