# Supplementary material for: Identification of the Sfp-Type PPTase EppA from the Lichenized Fungus Evernia prunastri
Source: PLoS One. 2016 Jan 19;11(1):e0145624. doi: 10.1371/journal.pone.0145624 (PMC4718654; doi:10.1371/journal.pone.0145624)
Supplement: S3 Table — (DOCX) [file pone.0145624.s003.docx]

**Supplementary table**

**S3 Table. Oligonucleotides used in this study.**

| **Oligonucleotide** | **5’-3’ sequence** |
| --- | --- |
| OS_SClys5_for | ACTAAAATGAGAATGAGGAGAAAGAGAATCACTCAGCAAAAAAACCGTGGGCAGGTCGACAACCCTTAAT |
| OS_SClys5_rev | AGAGTCTATCGATTACATAAATGTGAGCAAGCGAAAAAAAAAAATTGGCACCACTAGTGGATCTGATATCACCTA |
| OS_pYnpgA_for | TTAATATACCTCTATACTTTAACGTCAAGGAGAAAAAACCATGGTGCAAGACACATCAAG |
| OS_pYnpgA_rev | GCGGCCGCCAGTGTGATGGATATCTGCAGAATTCCAGCACTTAGGATAGGCAATTACACACC |
| OS_pYeppA_for | TTAATATACCTCTATACTTTAACGTCAAGGAGAAAAAACCCTGATCATGGAGGAGATAAAAGCCTTTCG |
| OS_pYeppA_rev | GCGGCCGCCAGTGTGATGGATATCTGCAGAATTCCAGCACTCACCCTCCTTGCCGGGGG |
| OS_SClys5_for | ACTAAAATGAGAATGAGGAGAAAGAGAATCACTCAGCAAAAAAACCGTGGGCAGGTCGACAACCCTTAAT |
| OS_SClys5_rev | AGAGTCTATCGATTACATAAATGTGAGCAAGCGAAAAAAAAAAATTGGCACCACTAGTGGATCTGATATCACCTA |
| OS_SClys5_a1 | CTTTAAAAGTGGAGCATCT |
| OS_SClys5_a2 | TACATATTGTTCACCGATG |
| OS_SClys5_a3 | CACTAATAGATTTTGGCGC |
| OS_SClys5_a4 | GTTCGCTGATACGTGTTAC |
| OS_SClys5_k2 | GGATGTATGGGCTAAATG |
| OS_SClys5_k3 | CAATCGTATGTGAATGCTG |
| OS_ck_for | AGGCGGTATTGCTTTCGTTG |
| OS_ck_rev | ATGTGTTTCCTGTGTGAAATTGTTATC |
| OS_ck_eppA_for | GGAATTGTGAGCGGATAACAATTTCACACAGGAAACACATATGGAGGAGATAAAAGCCTTTCGCTGG |
| OS_ck_eppA_rev | CGCAGGTCGGCCAACCAGTGCAACGAAAGCAATACCGCCTTCACCCTCCTTGCCGGGG |
