# Supplementary material for: Identification of the Sfp-Type PPTase EppA from the Lichenized Fungus Evernia prunastri
Source: PLoS One. 2016 Jan 19;11(1):e0145624. doi: 10.1371/journal.pone.0145624 (PMC4718654; doi:10.1371/journal.pone.0145624)
Supplement: S4 Table — (DOCX) [file pone.0145624.s004.docx]

**Supplementary table**

**S4 Table. Name and accession number of the protein sequences used in the phylogenetic tree.**

| **Name** | **Accession number** |
| --- | --- |
| *Nitrosococcus oceani* ATCC 19707_ABA57309 | ABA57309 |
| *Acinetobacter baumannii* ATCC 17978_ABO10606 | ABO10606 |
| *Actinobacillus succinogenes* 130Z_ABR74210 | ABR74210 |
| *Agrobacterium tumefaciens*_WP 025595094 | WP_025595094 |
| *Aspergillus fumigatus* var. RP-2014_KEY81881 | KEY81881 |
| *Aspergillus nidulans*_AAF12814 | AAF12814 |
| *Aspergillus niger* CBS 513.88_XP 001395469 | XP_001395469 |
| *Aspergillus oryzae*_XP 001826417 | XP_001826417 |
| *Bacillus subtilis*_WP 003224948 | WP_003224948 |
| *Bacillus subtilis*_WP 014112730 | WP_014112730 |
| *Bacillus subtilis*_WP 025709607 | WP_025709607 |
| *Bacillus subtilis*_WP 025709703 | WP_025709703 |
| *Bipolaris sorokiniana*_AER36018 | AER36018 |
| *Bordetella pertussis*_WP 010930724 | WP_010930724 |
| *Bradyrhizobium japonicum*_WP 018644576 | WP_018644576 |
| *Bradyrhizobium japonicum*_WP 018646187 | WP_018646187 |
| *Burkholderia pseudomallei*_WP 012729763 | WP_012729763 |
| *Burkholderia pseudomallei* S13_EDS82001 | EDS82001 |
| *Burkholderia pseudomallei* S13_EDS82021 | EDS82021 |
| *Campylobacter coli*_WP 002779165 | WP_002779165 |
| *Candida albicans*_AAO26020 | AAO26020 |
| *Citrobacter* sp. L17_EKU35973 | EKU35973 |
| *Coxiella burnetii* RSA 493_AAO89779 | AAO89779 |
| *Cystobacter fuscus* DSM 2262_EPX56039 | EPX56039 |
| *Cystobacter fuscus* DSM 2262_EPX64882 | EPX64882 |
| *Desulfobacterium autotrophicum* HRM2_ACN17368 | ACN17368 |
| *Escherichia coli*_KDO89827 | KDO89827 |
| *Evernia prunastri*_EppA | KT369532 |
| *Frateuria aurantia* DSM 6220_AFC84522 | AFC84522 |
| *Fusarium fujikuroi*_CCE73639 | CCE73639 |
| *Geobacter uraniireducens*_WP 011939195 | WP_011939195 |
| *Glomerella graminicola*_AAZ04409 | AAZ04409 |
| *Haemophilus influenzae*_WP 005658125 | WP_005658125 |
| *Haliangium ochraceum*_WP 012829463 | WP_012829463 |
| *Helicobacter pylori*_WP 021186553 | WP_021186553 |
| *Hirschia maritima*_WP 018996365 | WP_018996365 |
| *Janthinobacterium lividum*_EZP40290 | EZP40290 |
| *Klebsiella oxytoca*_WP 025107549 | WP_025107549 |
| *Legionella pneumophila* Paris_WP 010946521 | WP_010946521 |
| *Magnaporthe grisea* 70-15_XP 003720635 | XP_003720635 |
| *Marinobacter aquaeolei* VT8_ABM17261 | ABM17261 |
| *Methylobacterium extorquens*_WP 015822743 | WP_015822743 |
| *Myxococcus xanthus* DK 1622_ABF88504 | ABF88504 |
| *Myxococcus xanthus* DK 1622_ABF92179 | ABF92179 |
| *Nautilia profundicola*_WP 012663966 | WP_012663966 |
| *Neisseria gonorrhoeae*_KDN02368 | KDN02368 |
| *Neofusicoccum parvum* UCRNP2_XP 007588723 | XP_007588723 |
| *Nodularia spumigena* NSOR10_AAW67221 | AAW67221 |
| *Oscillatoria* PCC10802_WP 026098494 | WP_026098494 |
| *Pelobacter propionicus*_WP 011736125 | WP_011736125 |
| *Penicillium rubens* Wisconsin 54-1255_XP 002558841 | XP_002558841 |
| *Photorhabsdus asymbiotica*_CAQ83062 | CAQ83062 |
| *Photorhabdus asymbiotica*_WP 015833455 | WP_015833455 |
| *Pseudogymnoascus pannorum* VKM F-4514_KFY38884 | KFY38884 |
| *Pseudomonas aeruginosa* PAO1-VE13_AGY70480 | AGY70480 |
| *Psychromonas ingrahamii* 37_ABM04432 | ABM04432 |
| *Ralstonia solanacearum*_WP 021156115 | WP_021156115 |
| *Rickettsiella grylli*_EDP45735 | EDP45735 |
| *Saccharomyces cerevisiae*_CAA96866 | CAA96866 |
| *Saccharomyces cerevisiae* P283_EWH15743 | EWH15743 |
| *Salmonella enterica* LT2_AAL22443 | AAL22443 |
| *Schizosaccharomyces pombe*_CAA97348 | CAA97348 |
| *Schizosaccharomyces pombe*_CCD31324 | CCD31324 |
| *Shewanella woodyi* ATCC 51908_ACA87573 | ACA87573 |
| *Spongiibacter tropicus*_WP 022957217 | WP_022957217 |
| *Streptomyces albus*_WP 016575939 | WP_016575939 |
| *Streptomyces albus*_WP 016579169 | WP_016579169 |
| *Sulfurimonas gotlandica*_WP 008340362 | WP_008340362 |
| *Trichoderma virens* Gv29-8_EHK16960 | EHK16960 |
| *Verticillium alfalfae* VaMs.102_XP 003007446 | XP_003007446 |
| *Vibrio fischeri* ES114_AAW86578 | AAW86578 |
| *Wolinella succinogenes*_WP 011139756 | WP_011139756 |
| *Yersinia pestis* CO92_CAL20225 | CAL20225 |
